# Implications of Delayed Reopening in Controlling the COVID-19 Surge in Southern and West-Central USA

**DOI:** 10.34133/2021/9798302

**Published:** 2021-10-28

**Authors:** Raj Dandekar, Emma Wang, George Barbastathis, Chris Rackauckas

**Affiliations:** ^1^Department of Computational Science and Engineering, Massachusetts Institute of Technology, Cambridge, USA, MA 02139; ^2^Department of Electrical Engineering and Computer Sciences, Massachusetts Institute of Technology, Cambridge, MA 02139, USA; ^3^Department of Mechanical Engineering, Massachusetts Institute of Technology, Cambridge, MA 02139, USA; ^4^Singapore-MIT Alliance for Research and Technology (SMART) Centre, Singapore138602; ^5^Department of Applied Mathematics, Massachusetts Institute of Technology, Cambridge, MA 02139, USA

## Abstract

In the wake of the rapid surge in the COVID-19-infected cases seen in Southern and West-Central USA in the period of June-July 2020, there is an urgent need to develop robust, data-driven models to quantify the effect which early reopening had on the infected case count increase. In particular, it is imperative to address the question: How many infected cases could have been prevented, had the worst affected states not reopened early? To address this question, we have developed a novel COVID-19 model by augmenting the classical SIR epidemiological model with a neural network module. The model decomposes the contribution of quarantine strength to the infection time series, allowing us to quantify the role of quarantine control and the associated reopening policies in the US states which showed a major surge in infections. We show that the upsurge in the infected cases seen in these states is strongly corelated with a drop in the quarantine/lockdown strength diagnosed by our model. Further, our results demonstrate that in the event of a stricter lockdown without early reopening, the number of active infected cases recorded on 14 July could have been reduced by more than 40% in all states considered, with the actual number of infections reduced being more than 100,000 for the states of Florida and Texas. As we continue our fight against COVID-19, our proposed model can be used as a valuable asset to simulate the effect of several reopening strategies on the infected count evolution, for any region under consideration.

## 1. Background

The Coronavirus respiratory disease 2019 originating from the virus “SARS-CoV-2” [1, 2] has led to a global pandemic, leading to more than 50 million confirmed global cases in more than 200 countries as of November 13, 2020 [3]. In the United States, the first infections were detected in Washington State as early as January 20, 2020 [4], and now, it is being reported that the virus had been circulating undetected in New York City as early as mid-February [5]. As of September 21, 2020, the United States has ≈6.9 million infected cases since the virus began to spread.

**Figure 1:**
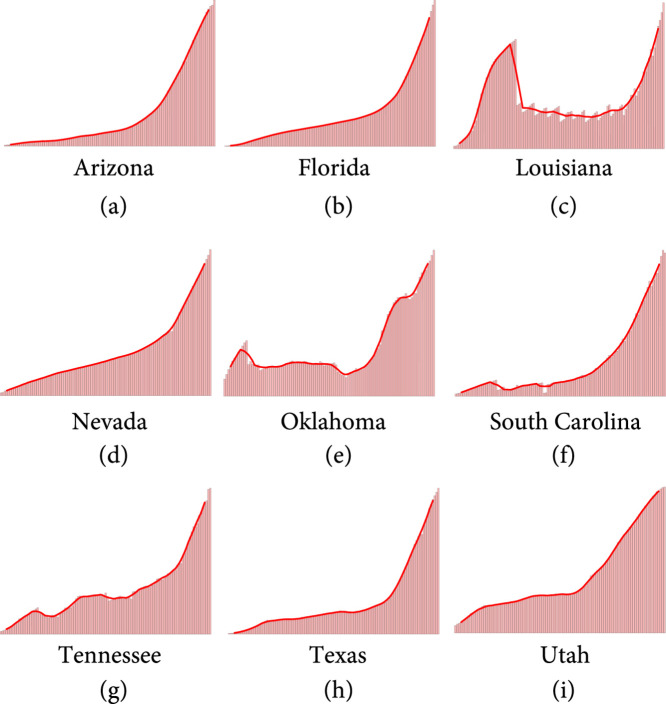
Active infected cases over time as of July 14, 2020, shown with a 7-day moving average, for the Southern and West-Central states considered in the present study.

Since the second week of June, a second surge of COVID-19 was seen in the United States [6], with rapidly increasing daily infected cases, hospitalization rates, and death rates [7, 8]. Initially driven by disastrous situations in the states of Arizona, South Carolina, Texas, Florida, and Georgia [6], the surge in cases was also later seen in several other Southern and West-Central states [9]. This surge can be seen in Figure 1 which shows the active infected cases over time as of July 14, 2020, with a 7-day moving average for 9 states. States which reopened early show a generally strong corelation with the rise in the infected cases over the 3-month period from late April to mid-July 2020 [9]. For example, states which opened before May 15 showed daily infected case increments as follows: Florida (1393%), Arizona (858%), South Carolina (999%), Alabama (547%), Oklahoma (477%), Tennessee (279%), Georgia (245%), Mississippi (215%), Nevada (697%), Texas (680%), and Utah (287%), while states which reopened after May 29 showed values as follows: Michigan (16%), Pennsylvania (−26%), New York (−52%), New Jersey (−32%), and Illinois (−54%). Thus, although early reopening seems to be corelated to the second surge of cases seen in the USA, there is a need for robust, data-driven quantification of the effect of early reopening on the growth of infected count data. More importantly, it is of utmost importance to answer the question: How many infected cases could have been prevented, had the worst affected states not reopened early? 

In an effort to address this question, we have developed a machine learning-aided epidemiological model. The novelty of our model arises from the fact that it allows us to decompose the contribution of quarantine/lockdown strength evolution to the infected data time series for the region under consideration. This enables us to simulate the effect of varying quarantine strength evolutions and hence varying reopening strategies on the infected count data. We define reopening as beginning when a state allows its stay-at-home order to expire or, in the case of states that never issued a stay-at-home order, when a state first starts allowing nonessential businesses, such as dine-in restaurants and hair salons, to reopen [10, 11]. The reopening details for the states considered in the study are shown in Table 1. Considering nine US states which showed a significant surge in cases since the last month, we demonstrate that our model shows a drop in the quarantine strength evolution when these states were reopened. Furthermore, we show that maintaining a strict lockdown without early reopening would have led to about 500,000 fewer infected cases in all these states combined.

**Table 1:** Reopening details for different states considered in the present study.

State	Reopening date	Reopening details
(1) Arizona	May 15	June 17: mask regulations strengthened
		June 29: partial reversal of reopening
(2) Florida	May 4	June 3: Phase 2 of reopening
(3) Louisiana	May 15	June 5: Phase 2 of reopening
(4) Nevada	May 9	May 26: Phase 2 of reopening
(5) Oklahoma	April 24	May 15: Phase 2 of reopening,
		June 1: Phase 3 of reopening
(6) South Carolina	May 4	May 4: Stay at home order lifted
		Further facilities reopened till May 18
(7) Tennessee	April 30	May 22: Phase 2 of reopening
(8) Texas	May 1	May 18: Phase 2 of reopening
		June 3: Phase 3 of reopening
(9) Utah	May 1	May 1: gradual reopening

## 2. Methods

### 2.1. QSIR Model

In general, neural networks with arbitrary activation functions are universal approximators [12– 14]. Unbounded activation functions in particular, such as the rectified linear unit (ReLU), have been known to be effective in approximating nonlinear functions with a finite set of parameters [15– 17]. Thus, a neural network solution is attractive to approximate quarantine effects in combination with analytical epidemiological models. The downside is that the internal workings of a neural network are difficult to interpret. The recently emerging field of scientific machine learning [18] exploits conservation principles within a universal differential equation [19], SIR in our case, to mitigate overfitting and other related machine learning risks. 

In the present work, the neural network is trained from publicly available infection and population data for COVID-19 for each state under study.

### 2.2. Standard SIR Model

The SIR (Susceptible-Infected-Recovered) is governed by the following set of ODEs:

(1)dSdt=−βStItN,(2)dIdt=βStItN−γIt,(3)dRdt=γIt,

where β and γ are the contact and recovery rates, respectively. We use this framework as our baseline model to be augmented with a neural network module. We do not consider the possibility of recovered individuals being reinfected [20]. We also do not consider the waning of immunity associated with COVID-19 as discovered in recent studies [21]. Here, β is the infection rate and γ is the recovery rate, and they are assumed to be constant in time. The total population $N=S\left(t\right)+I\left(t\right)+R\left(t\right)$ is seen to remain constant as well; that is, births and deaths are neglected. The recovered population is to be interpreted as those who can no longer infect others, so it also includes individuals who are deceased due to the infection. The possibility of recovered individuals to become reinfected is accounted for by SEIS models [20], but we do not use this model here, as the reinfection rate for COVID-19 survivors is considered to be negligible as of now. 

An important assumption of the SIR models is homogeneous mixing among the subpopulations. Therefore, this model cannot account for social distancing or social network effects. Additionally, the model assumes uniform susceptibility and disease progression for every individual, and that no spreading occurs through animals or other nonhuman means. Alternatively, the SIR model may be interpreted as quantifying the statistical expectations on the respective mean populations, while deviations from the model’s assumptions contribute to statistical fluctuations around the mean.

### 2.3. QSIR Model: ODE Formulation

The QSIR ODE model formulation is similar to the one studied previously [22] and is briefly explained in this section. The equations governing the QSIR model are as follows:

(4)dSdt=−βStItN,(5)dIdt=βStItN−γ+QtIt=βStItN−γ+NNW,UIt,(6)dRdt=γIt+δTt,(7)dTdt=QtIt−δTt=NNW,UIt−δTt.

The SIR model is augmented by introducing a time-varying quarantine strength rate term $Q\left(t\right)$ represented by a neural network [19] and a quarantined population $T\left(t\right)$, which is prevented from having any further contact with the susceptible population. Thus, the term $I\left(t\right)$ denotes the active infected population (Activelyinfected=Cumulativeinfected−Recovered) still having contact with the susceptibles, as done in the standard SIR model, while the term $T\left(t\right)$ denotes the infected population who are effectively quarantined and isolated. 

**Figure 2:**
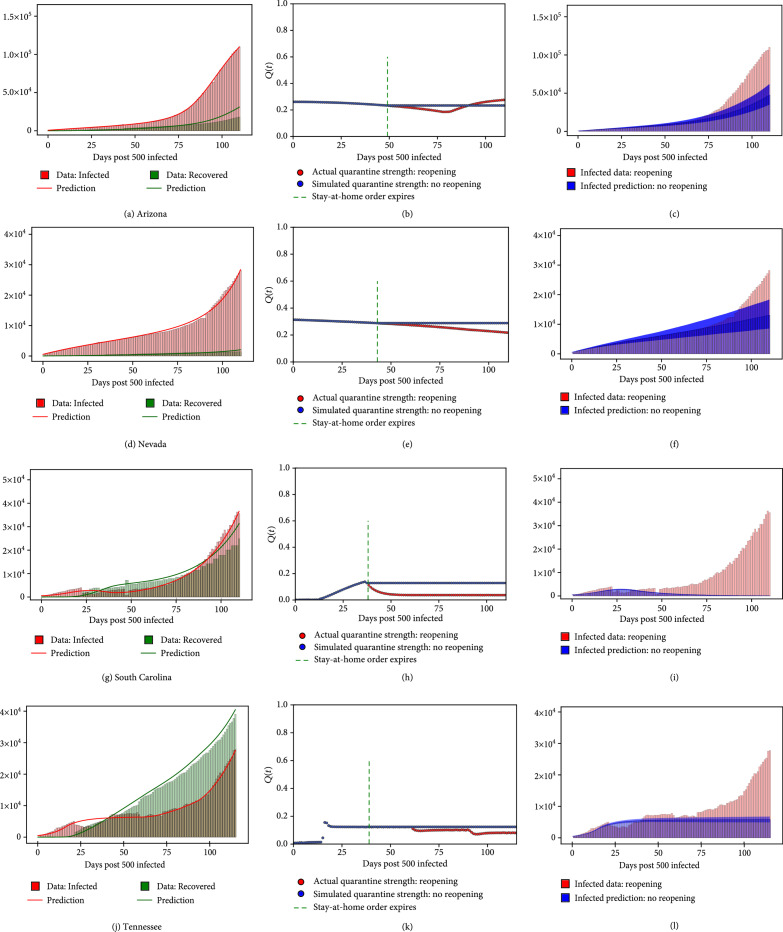
For the states of Arizona, Nevada, South Carolina, and Tennessee, the figure shows (a, d, g, and j) model recovery of infected and recovered case count as of 14 July 2020. (b, e, h, and k) Quarantine strength function as discovered by our trained model (with reopening). This is shown along with the quarantine strength function which we use to simulate strict quarantine without reopening after stay-at-home order was imposed. (c, f, i, and l) Estimated infected count if strict quarantine and lockdown measures were followed without reopening (5% and 95% quantiles are shown) as compared to the values corresponding to the actual early reopening scenario.

To study the effect of quarantine control, we start with the SIR epidemiological model. Figure 2(a) shows the schematic of the modified SIR model, the QSIR model, which we consider. We augment the SIR model by introducing a time-varying quarantine strength rate term $Q\left(t\right)$ and a quarantined population $T\left(t\right)$, which is prevented from having any further contact with the susceptible population. Thus, the term $I\left(t\right)$ denotes the active infected population (Activelyinfected=Cumulativeinfected−Recovered) still having contact with the susceptibles, as done in the standard SIR model, while the term $T\left(t\right)$ denotes the infected population who are effectively quarantined and isolated. Thus, we can write an expression for the quarantined infected population $T\left(t\right)$ as

(8)dTtdt=QtIt−δTt.

Since $Q\left(t\right)$ does not follow from first principles and is highly dependent on local quarantine policies, we devised a neural network-based approach to approximate it. 

Recently, it has been shown that neural networks can be used as function approximators to recover unknown constitutive relationships in a system of coupled ordinary differential equations [19, 23]. Following this principle, we represent $Q\left(t\right)$ as an n layer-deep neural network with weights $W_{1},W_{2}\cdots W_{n}$, activation function r, and the input vector $U=\left(S\left(t\right),I\left(t\right),R\left(t\right)\right)$ as

(9)Qt=rWnrWn−1⋯rW1U≡NNW,U.

**Figure 3:**
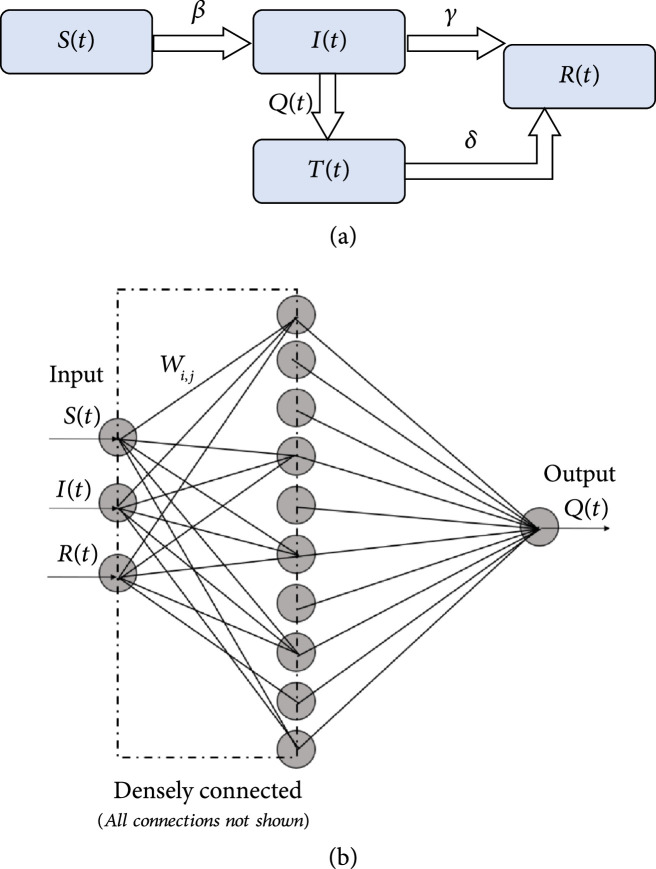
(a) Schematic of the augmented QSIR model considered in the present study. (b) Schematic of the neural network architecture used to learn the quarantine strength function $Q\left(t\right)$.

For the implementation, we choose a n=2-layer densely connected neural network with 10 units in the hidden layer and the leaky ReLU activation function. This choice was because we found sigmoidal activation functions to stagnate. The final model was described by 54 tunable parameters. The neural network architecture schematic is shown in Figure 3(b). The governing coupled ordinary differential equations for the QSIR model are

(10)dSdt=−βStItN,(11)dIdt=βStItN−γ+QtIt=βStItN−γ+NNW,UIt,(12)dRdt=γIt+δTt,(13)dTdt=QtIt−δTt=NNW,UIt−δTt.

### 2.4. Augmented QSIR Model: Initial Conditions

The starting point t=0 for each simulation was the day at which 500-infected cases was crossed, i.e., $I_{0}\approx 500$. The number of susceptible individuals was assumed to be equal to the population of the considered region. Also, in all simulations, the number of recovered individuals was initialized from data at t=0 as defined above. The quarantined population $T\left(t\right)$ is initialized to a small number $T\left(t=0\right)\approx 10$. 

### 2.5. Augmented QSIR Model: Parameter Estimation

The data for the infected, recovered case counts was obtained from the publicly maintained repository by the Center for Systems Science and Engineering at Johns Hopkins University. The loss function is defined as

(14)LNNW,β,γ,δ=logIt+Tt−logIdatat2+logRt−logRdatat2.

Parameter optimization for W,β,γ,andδ was performed by minimizing the loss function defined in Equation (14) using the approach employed in prior studies [22– 24] using an ADAM optimizer [25] with a learning rate of 0.01. For most of the states under consideration, W,β,γ,andδ were optimized by minimizing the loss function given in (14). For states with a low recovered count: Arizona, Florida, Nevada, and Texas, we employed a two-stage optimization procedure to find the optimal W,β,γ,andδ. In the first stage, (14) was minimized. For the second stage, we fix the optimal γ and δ found in the first stage to optimize for the remaining parameters: W,β based on the loss function defined just on the infected count as $L\left(W,\beta \right)={\left\|\log\left(I\left(t\right)+T\left(t\right)\right)-\log\left(I_{\text{data}}\left(t\right)\right)\right\|}^{2}$. Such an approach was found to be optimal for analyzing low recovered count data in previous studies [22]. 

In all states considered in the present study, we trained the model using data starting from the dates when the $500^{\text{th}}$ infection was recorded in each region and up to July 14, 2020. For each state considered, $Q\left(t\right)$ denotes the rate at which infected persons are effectively quarantined and isolated from the remaining population and thus gives composite information about (a) the effective testing rate of the infected population as the disease progresses and (b) the intensity of the enforced quarantine as a function of time. 

This QSIR ODE framework applied on the infected and recovered data is used to estimate the quarantine strength function $Q\left(t\right)$ in a particular state as shown in the first and second columns of Figure 2. 

### 2.6. QSIR Model: SDE Formulation

The ODE modelling framework described above is a deterministic approach to model transfer of species (here: people) from one compartment to another through different reaction channels. Such a deterministic approach ignores any random fluctuations during species transfer from one compartment to the other. To include such stochastic effects and thus get a measure of the model uncertainty, we note that the augmented SIR framework derives from the chemical master equation which describes the time evolution of the probability of such a system of interacting species to be in a given state at a given time (details in Supplementary Information (available here)). Although the chemical master equation cannot be solved analytically, under certain conditions, it can be distilled down to a stochastic differential equation (SDE) which captures the fluctuations in species transfer as random walks. Such an SDE, also known as the chemical Langevin Equation, is thus based on the underlying ODE framework (macroscopic picture) and also includes stochastic effects reminiscent of microscopic modelling. In fact, in the Supplementary Information, we show that the microscopic simulation, macroscopic ODE formulation, and chemical Langevin equation (which acts as a bridge between the two) are all equivalent to each other. 

The equivalent stochastic formulation or the chemical Langevin equation for the augmented SIR model is

(15)dS=−βStItNdt−βStItNdW1t,(16)dI=βStItN−γIt−QtItdt+βStItNdW1t−γItdW2t−QtItdW3t,(17)dR=γIt+δTtdt+γItdW2t+δTtdW4t,(18)dT=QtIt−δTtdt+QtItdW3t−δTtdW4t.

In (15), $W_{i}\left(t\right)\sim N\left(0,t\right)$ is a normally distributed random variable with mean zero and variance t or $dW_{i}\left(t\right)\sim N\left(0,dt\right)$. It should also be noted that each $W_{i}\left(t\right)$ represents an independent Brownian motion. The simulations were performed using the Catalyst.jl software in Julia using the LambaEM algorithm based on [26]. 1000 trajectories were simulated for each state. 

This QSIR SDE framework along with the simulated quarantine functions for no reopening is used to predict the new infected case count and hence estimate the reduction in the number of infected cases under the simulated no-reopening quarantine function. The results are shown as 5% and 95% quantiles in the third column of Figure 2. 

### 2.7. Mean Absolute Percentage Error

The Mean Absolute Percentage Error (MAPE) is defined as

(19)MAPE=100N∗∑It+Tt+Rt−Idatat+RdatatIdatat+Rdatat,

where N is the number of observations. 

## 3. Results

The first stage of our analysis is using our model [22], called the QSIR model to diagnose the underlying quarantine strength evolution $Q\left(t\right)$ in the regions under consideration. By applying the QSIR model to more than 70 countries globally, we have established the validity of $Q\left(t\right)$ in accurately diagnosing the on-the-ground quarantine situation in majorly affected European, South American, and Asian countries [22]. A slow growth of $Q\left(t\right)$ without a significant increase indicates relaxed quarantine policies, a sharp transition point in $Q\left(t\right)$ is indicative of a sudden ramp-up of quarantine measures, and an inflection point corresponds to the time when the quarantine response was the most rapid in the region under consideration. The results of our model applied globally to all continents are hosted publicly at http://covid19ml.org.

In this study, to perform the quarantine diagnosis to analyze the implications of delayed reopening, we applied the QSIR model to 9 US states which showed a significant surge in the infected case count in the last month: Arizona, Florida, Louisiana, Nevada, Oklahoma, South Carolina, Tennessee, Texas, and Utah. Figure 2 shows representative results for Arizona, Nevada, South Carolina, and Tennessee. The plots for the remaining states are provided in the Supplementary Information. Figures 2(a), 2(d), 2(g), and 2(j) show the comparison of the infected and recovered count estimated by our model with the actual data. A reasonable agreement is seen for all states, with the model being able to capture the rise in infections seen in the tail end of the time series. The QSIR model details are provided in Methods; Mean Absolute Percentage Error (MAPE) values for the model along with the epochs required for convergence for each state are provided in Supplementary Information.

Figures 2(b), 2(e), 2(h), and 2(k) show the quarantine strength evolution $Q\left(t\right)$ as learnt by the neural network module, which shows a decline whose starting point corresponds well to the time when these states began reopening, as seen from Table 2 and the green dotted line in Figures 2(b), 2(e), 2(h), and 2(k). In some states, the decline in $Q\left(t\right)$ starts later than the reopening date, possibly corresponding to the Phase 2 or Phase 3 of reopening (Table 2) or because of the time delay for population-level changes to be seen in the infected count evolution, after reopening. $Q\left(t\right)$ trained by our model shows a significant drop after early reopening in all Southern and West-Central states that showed a surge in cases last month, whereas the North-Eastern states of New York, New Jersey, and Illinois, which reopened late and showed no surge in infections, did not show a drop in $Q\left(t\right)$ (Table 3 and figures in Supplementary Information). Thus, the upsurge in the infected cases seen in these states is strongly corelated with a drop in the quarantine/lockdown strength $Q\left(t\right)$ diagnosed by our model. This is indicative of two things: (a) the Southern and West-Central states reopened early, which led to a relaxed imposition of quarantine/lockdown measures in these states and consequently a surge in infections was seen, and (b) the North-Eastern states of New York, New Jersey, and Illinois reopened late, and even after reopening, a relatively low contact rate was maintained among the population, leading to a relatively high magnitude of the imposed quarantine strength, which prevented a surge of infections in these states. The percentage decrease in quarantine strength observed after reopening for all states considered is shown in Table 3. It should be noted that for North-Eastern states which did not show a surge of infections last month, such as New York and New Jersey, such a drop in $Q\left(t\right)$ is not seen (figures in Supplementary Information). This indicates that the surge in infections, predominantly seen in the Southern and West-Central states, was caused by an early reopening which led to a relaxed imposition of quarantine/lockdown measures in these states.

**Table 2:** Drop in quarantine strength function,$Q\left(t\right)$, after reopening as discovered by our trained model. $Q\left(t\right)$ trained by our model shows a significant drop for all Southern and West-Central states which showed a surge in cases from reopening, whereas the North-Eastern states which showed no surge do not see a drop in $Q\left(t\right)$.

State	Reopening date	% increase in daily cases since reopening	Maximum % decrease in $Q\left(t\right)$ after reopening
(1) Arizona	May 15	+858	+22
(2) Florida	May 4	+1393	+10
(3) Louisiana	May 15	+193	+30
(4) Nevada	May 9	+697	+25
(5) Oklahoma	April 24	+477	+29
(6) South Carolina	May 4	+999	+71
(7) Tennessee	April 30	+279	+44
(8) Texas	May 1	+680	+29
(9) Utah	May 1	+287	+39
(10) New York	May 29	-52	-45
(11) New Jersey	June 9	-32	-60
(12) Illinois	May 29	-54	-8

**Table 3:** Infected count reduction by 14 July 2020, if states had not reopened early, as estimated by our model.

State	% decrease	Mean	Case reduction	Mean case
	(5%-95% quantiles)	% decrease		Case reduction
(1) Arizona	35-62	49	44000-79000	63000
(2) Florida	20-75	49	57000-218000	144000
(3) Louisiana	37-50	44	31000-41000	36000
(4) Nevada	32-68	51	10000-20000	15000
(5) Oklahoma	46-69	58	10000-15000	13000
(6) South Carolina	83-86	84	50000-52000	51000
(7) Tennessee	41-53	47	27000-36000	31000
(8) Texas	41-51	46	115000-143000	129000
(9) Utah	35-47	41	11000-14000	12000

To further demonstrate the validity of our model in capturing the actual quarantine policy evolution in a particular region, the model has been applied to 70 countries globally. The quarantine strength behaviour learnt from the model accurately mimics the on-the-ground situation in majorly affected European, South American, and Asian countries. The results of our model applied globally to all continents are hosted publicly at http://covid19ml.org.

After confirming that our model is able to accurately depict the corelation between the surge in infections and early reopening in these states through the diagnosed $Q\left(t\right)$, we proceed to the second stage of our analysis. In the second stage, we use the diagnosed $Q\left(t\right)$ to address the question: How many infected cases would have been reduced, had the worst affected states not reopened early? To answer this question, we simulate the “no-reopening” strategy by assuming that $Q\left(t\right)$ is maintained at the value it was before reopening, without decreasing. This simulated $Q\left(t\right)$ is shown in Figures 2(b), 2(e), 2(h), and 2(k). The flexibility of our model allows us to run our model with this simulated $Q\left(t\right)$ for all states considered. To quantify the aleatory uncertainty resulting from random fluctuations in the model, we utilized the chemical Langevin equation extension to the QSIR model whose definition and justification are described in Methods and Supplemental Information. This allows us to estimate bootstrapped confidence intervals resulting from 1000 simulations of such a stochastic model and thus quantify the effect of such a “no-reopening policy” on the epidemic spread. The infected count evolution for the simulated $Q\left(t\right)$ without reopening is shown in Figures 2(c), 2(f), 2(i), and 2(l) (5% and 95% quantiles are shown). We can see that, for all these states, instead of seeing a spike in infections, we would have seen a plateau in the infected case count evolution. The number and the percentage of infected cases that would have been prevented by July 14 had these states not reopened are shown in Table 3. It is evident that the number of infections could have been reduced by more than 40% in all states considered, with the actual number of infections reduced being more than 100,000 for the states of Florida and Texas. Even the less populated states of Louisiana, South Carolina, and Tennessee show mean infected case reduction values of 44%, 84%, and 47%, respectively, which correspond to 36,000, 51,000, and 31,000 infected cases reduced.

## 4. Conclusion

In this study, we have developed a novel methodology to quantify the effect of early reopening on the infected case count surge seen during the period of June-July 2020. We have proposed a machine learning model, called the QSIR model, rooted firmly in fundamental epidemiology principles which has the following attributes: (a) it is highly interpretable with few free parameters rooted in an epidemiological model, (b) it relies on only COVID-19 data and not on previous epidemics, and (c) it can decompose the infected time-series data to reveal the quarantine strength/policy variation, $Q\left(t\right)$, in the region under consideration. To demonstrate the validity of our model in capturing the actual quarantine policy evolution in a particular region, the model has been applied to 70 countries globally. The quarantine strength behaviour learnt from the model accurately mimics the on-the-ground situation in majorly affected European, South American, and Asian continents. The results for this global analysis are hosted at http://covid19ml.org [22].

After confirming our belief in the model through a global analysis, we apply the model to the Southern and West-Central US states which have shown a massive surge in COVID-19-infected cases since June 2020. We demonstrate that the $Q\left(t\right)$ extracted by our model shows a significant drop in value for the Southern and West-Central states which reopened early and showed a surge in infections. The time at which $Q\left(t\right)$ starts to decline generally agrees well with the reopening date for the states considered. Since the decline in $Q\left(t\right)$ is strongly corelated to the surge of infections and also the reopening date for states which reopened early, we can then simulate the effect of “no-reopening” by maintaining the $Q\left(t\right)$ at a constant level after reopening, instead of declining. We show that maintaining a steady imposition of quarantine/lockdown control would have played a massive role in bringing down the infected count by more than 40% in all states considered, with the infections reduced reaching more than 100,000 for the states of Florida and Texas.

We have proposed a novel machine learning methodology, rooted in fundamental epidemiological models, which is able to recover the real-time quarantine strength evolution for any region under consideration. As the pandemic evolves and we continue our fight against COVID-19, and for future outbreaks, our globally applicable methodology can be a valuable asset for researchers and policymakers to simulate several reopening strategies and counterfactual scenarios and analyze their impact on the infected count evolution. Our findings highlight that as we continue the fight against COVID-19, it is imperative to reduce the contact between susceptible and infected individuals in public places by formulating robust safety guidelines. Such guidelines implemented and maintained in the affected states would ensure a high level of quarantine strength associated with that state and can prevent a future surge or wave in the COVID-19-infected count time series.

Validation of the model robustness and parameter identifiability have been mentioned in the Supplementary Information. We have also compared an equivalent of the effective reproduction number called the COVID spread parameter in our study, with other studies to further validate the results of our modelling approach. The COVID spread parameter is defined by (a) the infected individuals and (b) the recovered individuals from both the infected and the quarantined states, since both of those effectively do not further contribute to the infection spread [22].

The results of our model should be taken in the context of its assumptions. Ideally, one needs to consider the shifting US testing policies for the time period under consideration. Since the testing efforts did not show a significant increase during and after the reopening in the US states in the time period considered within the present study [27, 28] and we did not want to burden our model with additional parameters to fit, testing compartments have not been included in the present study. Additionally, several studies in literature [29– 32] have attempted to incorporate underreporting of infected/recovered cases in their modelling paradigm. Most of these studies use previously known estimates of testing data, serology data, or Infection-Fatality-Rate (IFR). In these studies involving multiple parameters, a number of parameters are assumed to be fixed at the start of the simulation from prior studies. These parameters include and are not limited to time between onset of infections and symptoms, transmission duration, rate at which hospitalized patients recover [32], mean duration from symptom onset to recovery [29], or even the IFR ratio [29]. A second class of studies uses antibody testing from collected serum samples to estimate the actual number of infected cases [33].

As the pandemic unfolds and starts spreading, the first information available is the number of infected, recovered, and deaths (for example, the Johns Hopkins public repository for COVID-19 tracking). Unless we have serum sample data information or we can confidently rely on prior studies for assessment of certain parameters, accurate information of the underreporting factor is difficult to obtain in real time. One of the goals of the present modelling methodology is to assist researchers and policymakers with quarantine diagnosis information in real time, with no reliance on parameters derived from prior studies.

Finally, the model is based on the SIR framework, which assumes a constant, age-independent contact and recovery rate between the infected and susceptible populations. Additionally, we do not consider the spatial heterogeneity in the infected count within a particular state and assume the governing dynamics to be only time-dependent. Consideration of these second-order aspects would further refine the model and would be the subject of future studies.

Determining the optimal reopening policy for different states is a composite challenge depending on a wide range of social, economic, and political factors beyond the scope of the present study. Our results show that irrespective of these factors and their role in influencing the reopening policy, it is imperative to reduce the contact rate between infected and susceptible individuals, thereby maintaining or increasing the quarantine strength. When a state reopens its public spaces like restaurants, bars, schools, and cinema halls, the state reduces its quarantine strength, and even a small drop in this number can be enough to lead to a massive surge in the infected count. When a state has to reopen due to socioeconomic or political factors, it should do so with the utmost care and with detailed guidelines for reducing the contact rate as much as possible in schools, child care programs, offices, restaurants, bars, and vehicles of mass transit. This aligns well with the COVID-related safety guidelines issued by the CDC [34].

## Data Availability

Data for the infected and recovered case count in all regions was obtained from the Center for Systems Science and Engineering (CSSE) at Johns Hopkins University. All code files and results are publicly available at https://github.com/RajDandekar/Reopening_ImpactSimulator_US_States.

## Additional Points

*Resource Availability*. Lead contact is Raj Dandekar, MIT: Email: rajd@mit.edu.

## Supplementary Materials

Supplementary Materials Model-diagnosed quarantine strength for North-Eastern US state. Impact of early reopening on the states of Louisiana, Florida, Oklahoma, Texas, and Utah. Equivalence between the ODE model and the chemical Langevin SDE model. Model specifications for each state. Parameter inference: Gaussian process residue model. Model validation: calculation of the effective reproduction number.
