# Supplementary material for: Implications of Delayed Reopening in Controlling the COVID-19 Surge in Southern and West-Central USA
Source: Health Data Sci. 2021 Oct 28;2021:9798302. doi: 10.34133/2021/9798302 (PMC9629682; doi:10.34133/2021/9798302)
Supplement: Supplementary Materials — Model-diagnosed quarantine strength for North-Eastern US state. Impact of early reopening on the states of Louisiana, Florida, Oklahoma, Texas, and Utah. Equivalence between the ODE model and the chemical Langevin SDE model. Model specifications for each state. Parameter inference: Gaussian process residue model. Model validation: calculation of the effective reproduction number. [file 9798302.f1.pdf]

Supplementary Information: Implications of delayed reopening in controlling the COVID-19 surge in Southern and West-Central USA

Raj Dandekar[1, 6, *], Emma Wang[2], George Barbastathis[3, 4], and Chris Rackauckas[5]

[1]Department of Computational Science and Engineering, Massachusetts Institute of Technology, Cambridge, MA 02139, USA
[2]Department of Electrical Engineering and Computer Sciences, Massachusetts Institute of Technology, Cambridge, MA 02139, USA
[3]Department of Mechanical Engineering, Massachusetts Institute of Technology, Cambridge, MA 02139, USA
[4]Singapore-MIT Alliance for Research and Technology (SMART) Centre, Singapore 138602
[5]Department of Applied Mathematics, Massachusetts Institute of Technology, Cambridge, MA 02139, USA
[6]Lead Contact
[*]Correspondence: rajd@mit.edu

September 14, 2021

Supplementary Information

Model-diagnosed quarantine strength for North-Eastern US states

Figure S1 shows the application of the model to the north-eastern states of New York, New Jersey and Illinois along with the diagnosed quarantine strength function $Q(t)$ for these states. These states do not show a decline in $Q(t)$. This corresponds well to the delayed reopening and generally stronger quarantine measures employed in the North-Eastern US states. Since $Q(t)$ does not decrease, these states did not show a surge in infections starting June 2020, unlike their Southern and West-Central counterparts. The difference in these results between the North-Eastern and Southern, West-Central states indicates two things: (a) it strengthens the validity of our proposed model in capturing the real-time reopening scenario in different states through the evolution of the diagnosed $Q(t)$, and, more importantly, (b) it further validates the role played by early reopening in reducing $Q(t)$ and subsequently leading to a surge of new infected cases in the Southern and West-Central US states.

Impact of early reopening on the states of Louisiana, Florida, Oklahoma, Texas and Utah

Figure S2, S3 implements a similar analysis to study the effect of early reopening for the states of Louisiana, Nevada, Oklahoma, Texas and Utah, as done for the states of Arizona, Nevada, South Carolina and Tennessee. Similar to the states considered in the main text, we see that all of these states show a decline in $Q(t)$ starting around the time when these states were reopened. If these states were not reopened early, a large number of infections would have been reduced as demonstrated in Table 3 of the main text.

Equivalence between the ODE model and the Chemical Langevin SDE model

This analysis heavily borrows from the pioneering work done by Gillespie.[1] In this section, we will establish that the deterministic ODE model and the stochastic Chemical Langevin equation originate from a common expression: the chemical master equation,[2] and are closely linked to one another. Following is the notation we will use, in accordance with[1] We consider N compartments: $S_1, S_2 \dots S_N$ and R reaction channels: $R_1, R_2 \dots R_M$ in a fixed volume Ω. In our case, we have $N = 4$ (S, I, R, T) compartments and $R = 4$ reaction channels. We denote the dynamical state of the system at any time t as $X(t) = (X_1(t), X_2(t) \dots X_N(t))$ where

- $X_i(t)$: total number of S_i molecules (in our case: individuals) in the system.
- Propensity function $a_j(x)dt$: probability that a reaction R_j will occur somewhere in Ω in the next time interval [t, t+dt] for $j = 1, 2 \dots M$.
- State change vector ν_j whose ith component is defined by $\nu_{j,i}$: change in the number of S_i molecules produced by one R_j reaction for $i = 1, 2 \dots N$, $j = 1, 2 \dots M$. In our case $\nu_{j,i} = \pm 1$.

From the definition of $a_j(x)dt$, we can write the probability of the system being in state x at time $t + dt$ (we take the sum of all mutually exclusive ways either through one reaction or no reaction in [t, t+dt]):

$$P(x, t + dt | x_0, t_0) = P(x, t | x_0, t_0) \left[1 - \sum_{j=1}^{M} a_j(x) dt \right] + \sum_{j=1}^{M} \left[P(x - \nu_j, t | x_0, t_0) a_j(x - \nu_j) dt \right], \tag{1}$$

Taking the limit of (1) as dt -¿ 0 leads to the **chemical master equation**

$$\frac{\partial P(x, t | x_0, t_0)}{\partial t} = \sum_{j=1}^{M} \left[a_j(x - \nu_j) P(x - \nu_j, t | x_0, t_0) - a_j(x) P(x, t | x_0, t_0) \right] \tag{2}$$

.

Macroscopic picture: Deterministic model relation to the chemical master equation:

Multiplying the chemical master equation (2) by x_i and summing over all x, we obtain for the mean of $X_i(t)$

$$\frac{d\langle X_i(t) \rangle}{dt} = \sum_{j=1}^{M} \nu_{ji} \langle a_j(X(t)) \rangle \qquad (i = 1, 2 \dots N) \tag{3}$$

Thus, whenever fluctuations are not important, the species populations evolve deterministically according to the following set of ordinary differential equations

$$\frac{dX_i(t)}{dt} = \sum_{j=1}^{M} \nu_{ji} a_j(X(t)) \qquad (i = 1, 2 \dots N) \tag{4}$$

(4) is the basis for the classical SIR epidemiological equations, and we see how they evolve from the chemical master equation (2).

$$\frac{dS}{dt} = -\frac{\beta\, S(t)\, I(t)}{N} \tag{5}$$

$$\begin{aligned} \frac{dI}{dt} &= \frac{\beta\, S(t)\, I(t)}{N} - (\gamma + Q(t))\, I(t) \\ &= \frac{\beta\, S(t)\, I(t)}{N} - (\gamma + \mathrm{NN}(W, U))\, I(t) \end{aligned} \tag{6}$$

$$\frac{dR}{dt} = \gamma I(t) + \delta T(t) \tag{7}$$

$$\frac{dT}{dt} = Q(t)\, I(t) - \delta T(t) = \mathrm{NN}(W, U)\, I(t) - \delta T(t). \tag{8}$$

The ODE system used in the present study shown in (5-8), is of the form (4).

Microscopic picture: Stochastic Simulation Algorithm and its relation to the master equation:

Another consequence of the master equation (2) is the existence and form of the next-reaction density function $p(\tau, j|x, t)$, which is defined as

- $p(\tau, j|x, t)d\tau$ = probability that given $X(t) = x$, the next reaction in Ω will occur in $[t+\tau, t+\tau+d\tau]$, and will be an R_j reaction

Since $\sum_j a_j(x)dt$ is the probability that some reaction occurs in the time interval dt, the probability that a time interval τ is spent without any reaction occuring is given by the exponential distribution: $\text{Exp}(\sum_j a_j(x)\tau)$. Thus, we obtain for $p(\tau, j|x, t)$

$$p(\tau, j|x, t) = a_j(x)\text{Exp}\left(\sum_{k=1}^{M} a_k(x)\tau\right) \qquad (0 \le \tau < \infty; j = 1, 2 \ldots M) \tag{9}$$

(9) is the basis for the stochastic simulation algorithm in which Monte-Carlo techniques are used to construct unbiased realizations of the process $X(t)$. A typical algorithm for stochastic simulation of this kind, is the Gillespie Algorithm[3] which can be viewed as a discrete space continuous time Markov jump process, with exponentially distributed jump times.

Chemical Langevin Equation: Bridging the gap between macroscopic and microscopic models:

Let the state of the system $X(t)$ at the current time t be x_t. Let $K_j(x_t, \tau)$ be the number of R_j reactions that occur in the time interval [t, t+dt]. Thus, the number of S_i molecules in the system at time $t + \tau$ will be

$$X_i(t+\tau) = x_{ti} + \sum_{j=1}^{M} K_j(x_t, \tau)\nu_{ji} \qquad (i = 1, 2 \ldots N) \tag{10}$$

[1] approximated K_j by imposing the following conditions

- **Condition 1: No propensity function change** This condition requires τ to be small enough so that none of the propensity functions $a_j(x)$ change noticeably. The propensity functions then satisfy

$$a_j(X(t')) \approx a_j(x_t) \qquad \forall t \in [t, t+\tau], \forall j \in [1, M] \tag{11}$$

  Due to this condition, $K_j(x_t, \tau)$ will be a statistically independent Poisson random variable $P_j(a_j(x_t), \tau)$. Thus (10) simplifies to

$$X_i(t+\tau) = x_{ti} + \sum_{j=1}^{M} \nu_{ji} P_j(a_j(x_t), \tau) \qquad (i = 1, 2 \ldots N) \tag{12}$$

- **Condition 2: Large number of reaction occurrences:** This condition requires τ to be large enough so that the expected number of occurrences of each reaction channel R_j in $[t, t+\tau]$ is much larger than 1. Thus

$$\langle P_j(a_j(x_t), \tau) \rangle = a_j(x_t)\tau \gg 1, \qquad \forall j \in [1.M]. \tag{13}$$

  This condition enables us to approximate each Poisson variable $P_j(a_j(x_t), \tau)$ by a normal random variable with the same mean and variance.

  Thus, (12) further simplifies to

$$X_i(t+\tau) = x_{ti} + \sum_{j=1}^{M} \nu_{ji} N_j(a_j(x_t)\tau, a_j(x_t)\tau) \qquad (i = 1, 2 \ldots N) \tag{14}$$

where $N(m,\sigma^2)$ denotes the normal random variable with mean m and variance σ^2. Using $N(m,\sigma^2) = m + \sigma N(0,1)$, denoting the time interval τ by dt and the unit normal random variable $N_j(0,1)$ as $N_j(t)$, we obtain

$$X_i(t+dt) = X_i(t) + \sum_{j=1}^{M} \nu_{ji} a_j(X(t))dt + \sum_{j=1}^{M} \nu_{ji} a_j^{1/2}(X(t))N_j(t)(dt)^{1/2} \qquad (i = 1,2\ldots N) \quad (15)$$

(15) can be written as a stochastic differential equation as

$$\frac{dX_i(t)}{dt} = \sum_{j=1}^{M} \nu_{ji} a_j(X(t)) + \sum_{j=1}^{M} \nu_{ji} a_j^{1/2}(X(t))\Gamma_j(t) \quad (16)$$

where $\Gamma_j(t)$ are temporally uncorrelated, statistically independent Gaussian white noise processes.

(16) is the Langevin equation, and it derives from the master equation provided that Condition 1 and Condition 2 are satisfied.

The Langevin equation (16) form of the ODE system (5-8) leads to the stochastic differential equation used in the current study

$$dS = -\left[\frac{\beta\, S(t)\, I(t)}{N}\right] dt - \sqrt{\left[\frac{\beta\, S(t)\, I(t)}{N}\right]} dW_1(t) \quad (17)$$

$$dI = \left[\frac{\beta\, S(t)\, I(t)}{N} - \gamma I(t) - Q(t)I(t)\right] dt + \sqrt{\frac{\beta\, S(t)\, I(t)}{N}} dW_1(t) - \sqrt{\gamma I(t)} dW_2(t) - \sqrt{Q(t)I(t)} dW_3(t) \quad (18)$$

$$dR = [\gamma I(t) + \delta T(t)]\, dt + \sqrt{\gamma I(t)} dW_2(t) + \sqrt{\delta T(t)} dW_4(t) \quad (19)$$

$$dT = [Q(t)\, I(t) - \delta T(t)]\, dt + \sqrt{Q(t)I(t)} dW_3(t) - \sqrt{\delta T(t)} dW_4(t) \quad (20)$$

In (17), $W_i(t) \sim N(0,t)$ is a normally distributed random variable with mean zero and variance t or $dW_i(t) \sim N(0,dt)$. It should also be noted that each $W_i(t)$ represents an independent Brownian motion.

Comparison of the macroscopic, microscopic and Langevin SDE model for our study

Figure S4a shows that the microscopic Stochastic Simulation Gillespie Algorithm and the ODE model presented in Equation (6-9) in the main text show a good agreement with each other. Figure S4b shows the comparison of the Chemical Langevin SDE model shown in (17) ran for 1000 trajectories and the ODE model; which also show a good agreement. Thus, we have shown the equivalence between the microscopic, macroscopic and the Chemical Langevin model for our study. This equivalence allows us to add fluctuating components to the standard deterministic SIR model as shown in (17) and quantify the uncertainty resulting from these fluctuations.

Model specifications for each state

Table S1 shows the Model Mean Absolute Percentage Error (MAPE), epochs needed for convergence and number of parameters optimized for the different states considered.

Parameter Inference: Gaussian Process Residue Model

In order to validate the robustness of the model and the uniqueness of the parameters recovered by the model, we consider a Gaussian Process residue model for uncertainty quantification. Gaussian Processes have emerged as a useful tool for regression, classification, clustering and uncertainty quantification.[4,5]

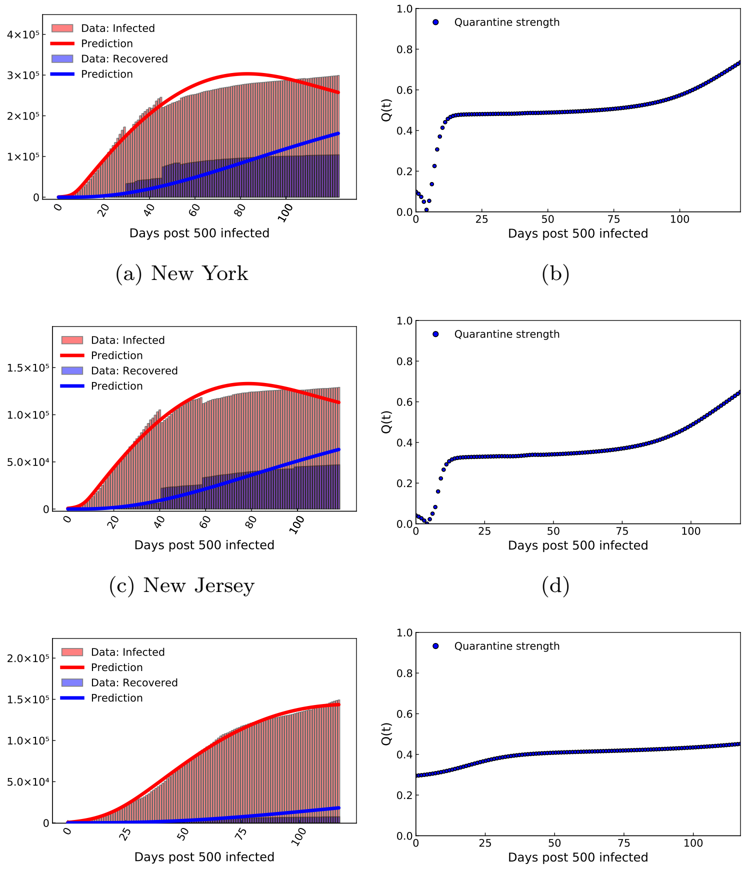

(a) New York (b)

(c) New Jersey (d)

(e) Illinois (f)

Figure S1: For the states of New York, New Jersey and Illinois, figure shows: (a, c, e) Model recovery of infected and recovered case count trained until 14 July, 2020. (b, d, f) Quarantine strength function as discovered by our trained model

Table S1: Mean Absolute Percentage Error (MAPE) values are shown along with the number of epochs required for and the number of parameters optimized, for all states considered.

State	Model MAPE	Epochs	Parameters optimized
1. Arizona	5.4%	10^5	54
2. Florida	18.7%	10^5	54
3. Louisiana	12%	12^5	54
4. Nevada	3.14%	18^5	54
5. Oklahoma	7.9%	12^5	54
6. South Carolina	11.7%	12^5	54
7. Tennessee	6.9%	12^5	54
8. Texas	10.4%	24^5	54
9. Utah	3.79%	12^5	54

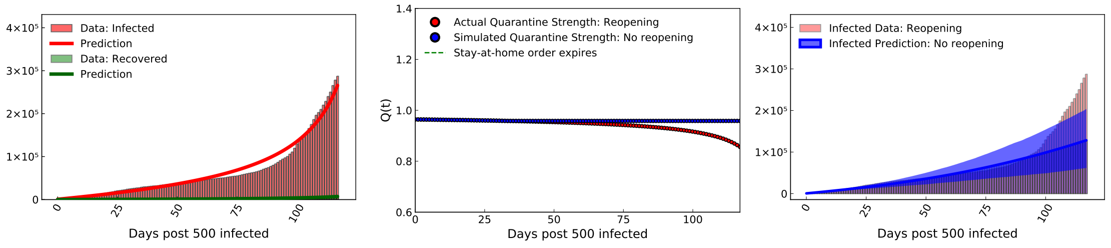

(a) Florida (b) (c)

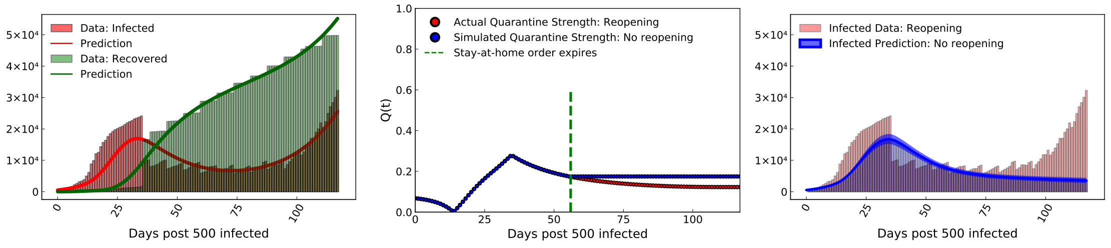

(d) Louisiana (e) (f)

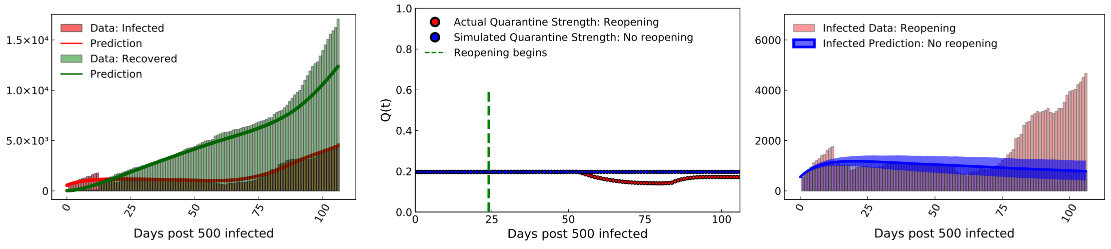

(g) Oklahoma (h) (i)

Figure S2: For the states of Louisiana, Nevada and Oklahoma: (a, d, g) Model recovery of infected and recovered case count as of 14 July, 2020. (b, e, h) Quarantine strength function as discovered by our trained model (with reopening). This is shown along with the quarantine strength function which we use to simulate strict quarantine without reopening after stay-at-home order was imposed. (c, f, i) Estimated infected count if strict quarantine and lockdown measures were followed without reopening as compared to the values corresponding to the actual early reopening scenario.

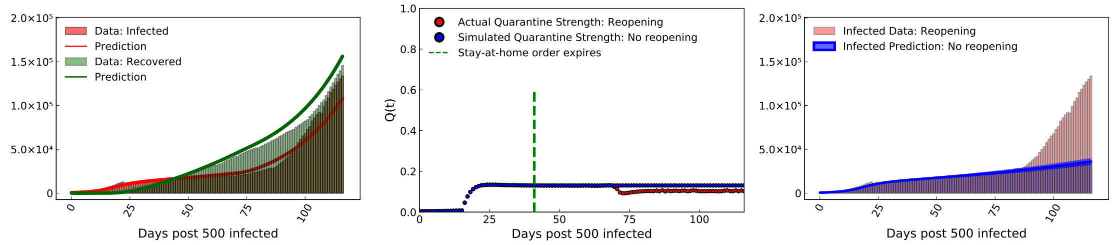

(a) Texas (b) (c)

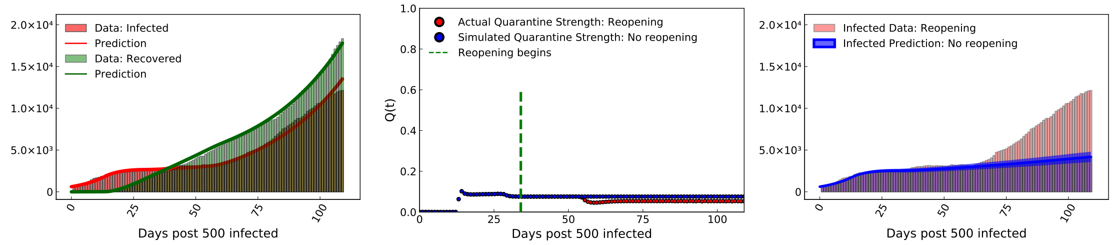

(d) Utah (e) (f)

Figure S3: For the states of Texas and Utah: (a, d) Model recovery of infected and recovered case count as of 14 July, 2020. (b, e) Quarantine strength function as discovered by our trained model (with reopening). This is shown along with the quarantine strength function which we use to simulate strict quarantine without reopening after stay-at-home order was imposed. (c, f) Estimated infected count if strict quarantine and lockdown measures were followed without reopening as compared to the values corresponding to the actual early reopening scenario.

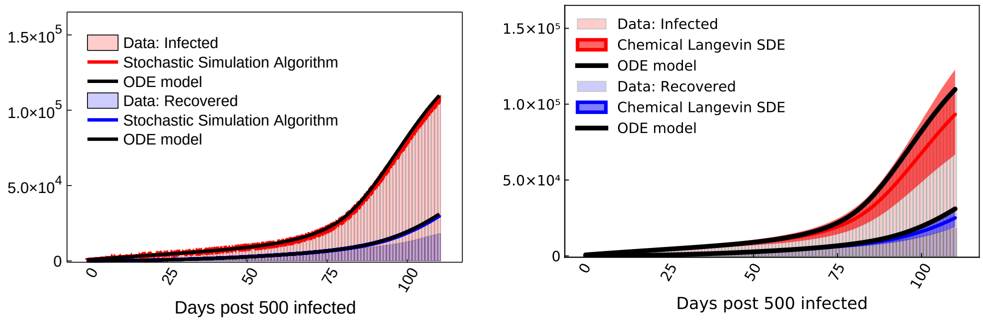

(a) Arizona (b)

Figure S4: (a) Comparison of the microscopic Stochastic Simulation Gillespie Algorithm and the ODE model presented in Equation (6-9) in the main text. (b) Comparison of the Chemical Langevin SDE model shown in (17) ran for 1000 trajectories ($5\% and 95\%$ quantiles are shown) and the ODE model.

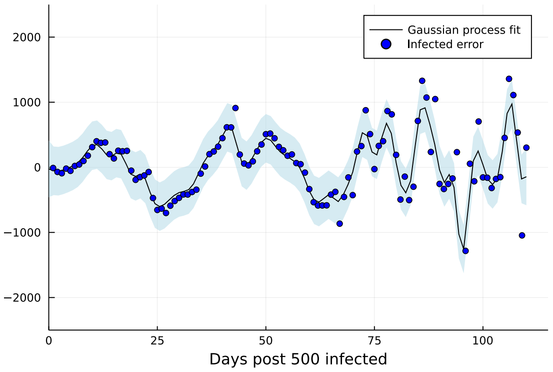

Figure S5: [Gaussian Process Residue Regression Model] Gaussian Process residue model fitted to the infected case count shown for Arizona.

In the present study, we fit a Gaussian Process regression model between the error resulting from the best fit model and the infected data. For the prior over the function space, we use a mean of zero and variance described by a Squared Exponential Kernel with a lengthscale of 1 and a significantly high signal standard deviation of $O(10^4)$ which allows for noisy estimates of the posterior. Such a fitted model for the infected count for a region under consideration (Arizona), is shown below in figure S5. Subsequently, we sample 500 error residues from this model and superimpose them on the best fit predictions to simulate 500 samples of the infected case count data. Finally, we apply our model described on these 500 samples of data, and recover the parameters $Q(t), \beta, \gamma, \delta$ from each of them.
Figures S6, S7 shows the inferred parameters for 500 realizations of the Gaussian process residue model superimposed on the best fit model prediction applied to all states considered, and shown for (a) the quarantine strength function $Q(t)$, (b) the contact rate β and (c) the recovery rate $\gamma+\delta$. It can be seen that for all realizations, $Q(t)$ is seen to follow a similar behaviour, which lies close to the best fit model prediction. In addition, the inferred histograms for the contact rate β and the recovery rate $\gamma + \delta$ show a peak which is close to the best fit model prediction. This further validates the robustness of the model for other regions considered and strengthens the uniqueness of the parameters recovered by the model. A total of 12 million iterations (60000 iterations for each realization of the Gaussian process residue model × 500 realizations) were performed on the MIT Supercloud cluster to generate parameter histograms for each state considered.

Model validation: Calculation of the effective reproduction number

Following a previous study,[6] we define the Covid spread parameter as follows:

$$C_p(t) = \left[\frac{\beta}{Q(t) + \gamma + \delta}\right] S(t)/N \tag{21}$$

where $S(t)$ is the susceptible population and N is the total population. This definition of the Covid spread parameter $C_p(t)$ is equivalent to the effective reproduction number $R_{eff}(t)$ in the context of the QSIR model. We included both γ, δ in the definition of $C_p(t)$ since both these parameters eventually contribute to the recovered population and we wanted to include effects of both. Another viable option to define $C_p(t)$ could be to just use γ in the denominator of $C_p(t)$.

Figure S8 shows the comparison of the Covid spread parameter, as defined in Equation 21 with and without reopening for all US states considered in the present study. For all the states, we can see that without reopening, a diminished effective reproduction number is seen, indicating moving in the right direction of halting the infection spread.

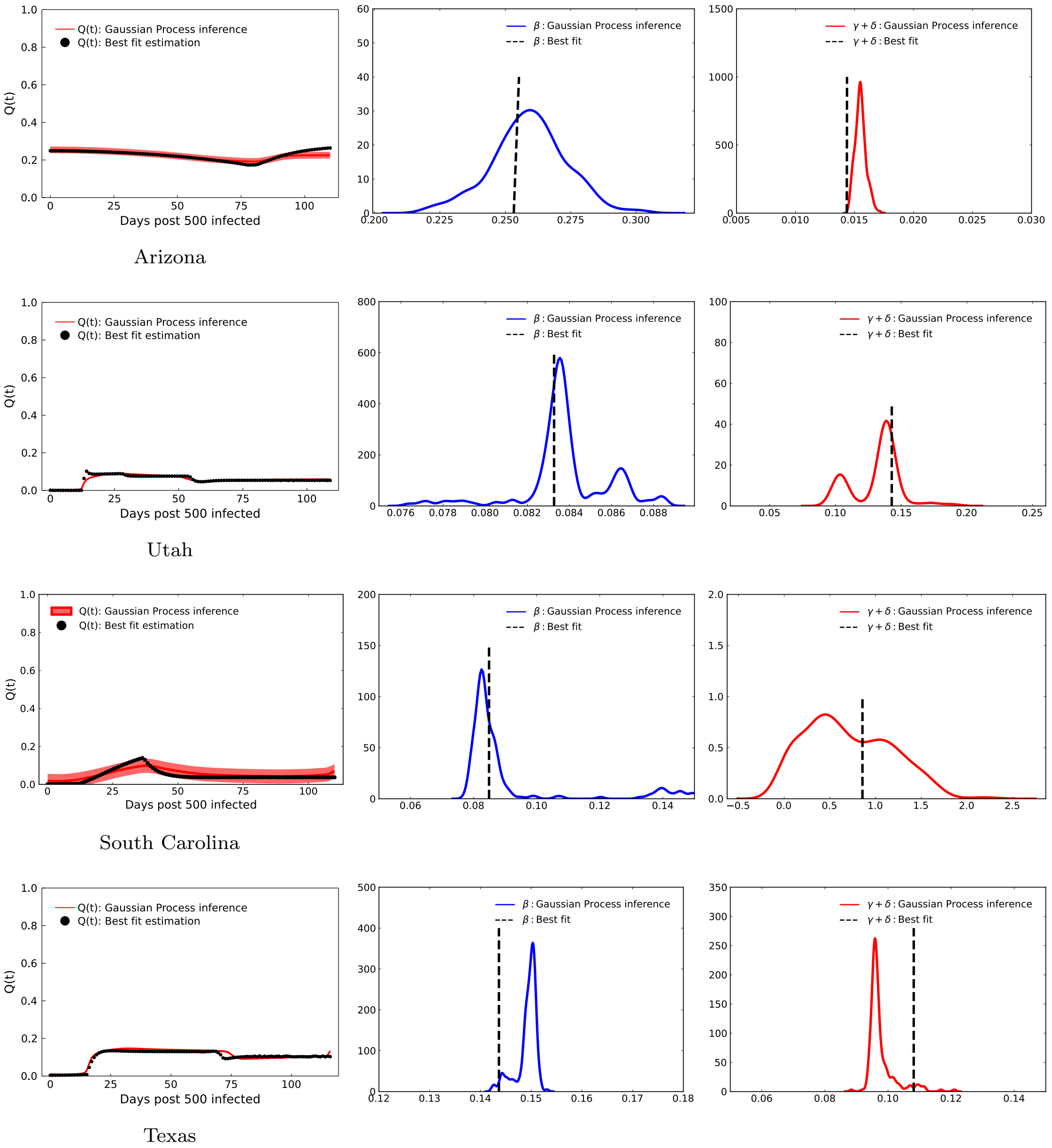

Figure S6: [Parameter Inference for US states] Inferred parameters for 500 realizations of the Gaussian process residue model superimposed on the best fit model prediction applied to the region considered for demonstration, and shown for (a) the quarantine strength function $Q(t)$, (b) the contact rate β and the recovery rate $\gamma + \delta$. A total of 12 million iterations were performed on the MIT Supercloud cluster to generate parameter histograms for one state.

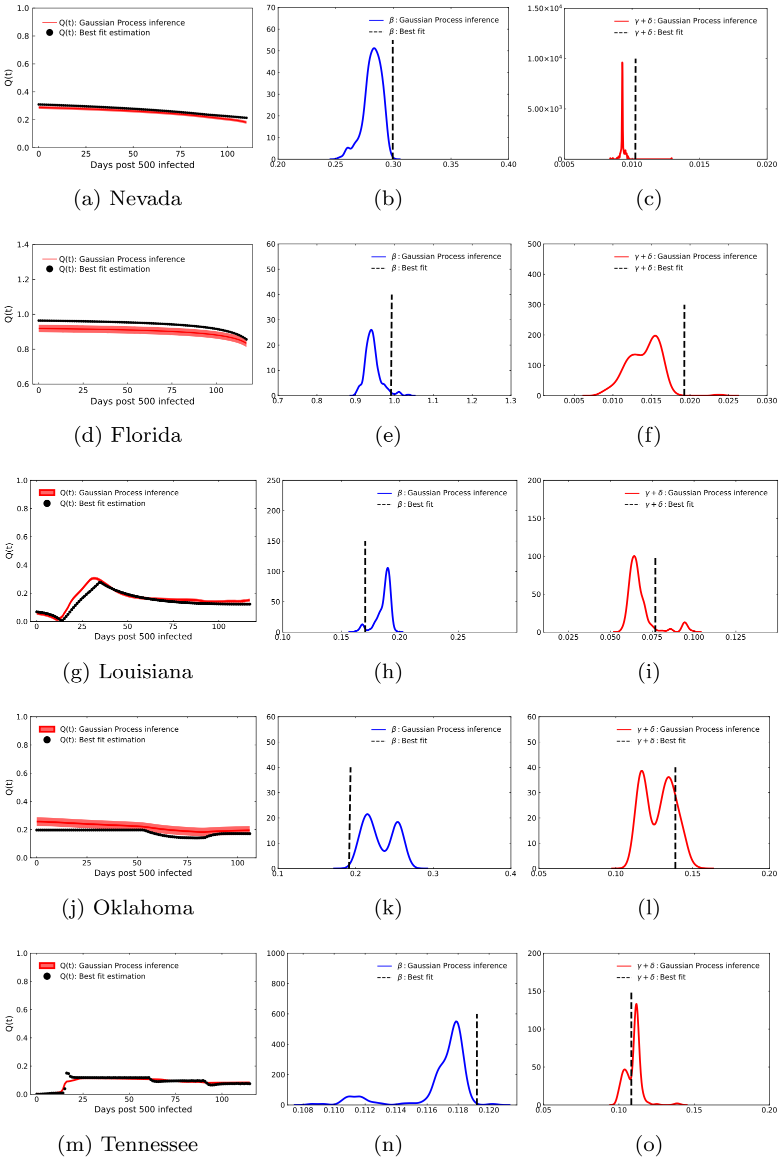

Figure S7: [Parameter Inference for US states] Inferred parameters for 500 realizations of the Gaussian process residue model superimposed on the best fit model prediction and shown for the quarantine strength function $Q(t)$ (left column), the contact rate β (middle column) and the recovery rate $\gamma + \delta$ (right column) for the US states considered in the present study. A total of 12 million iterations were performed on the MIT Supercloud cluster to generate parameter histograms for each region.

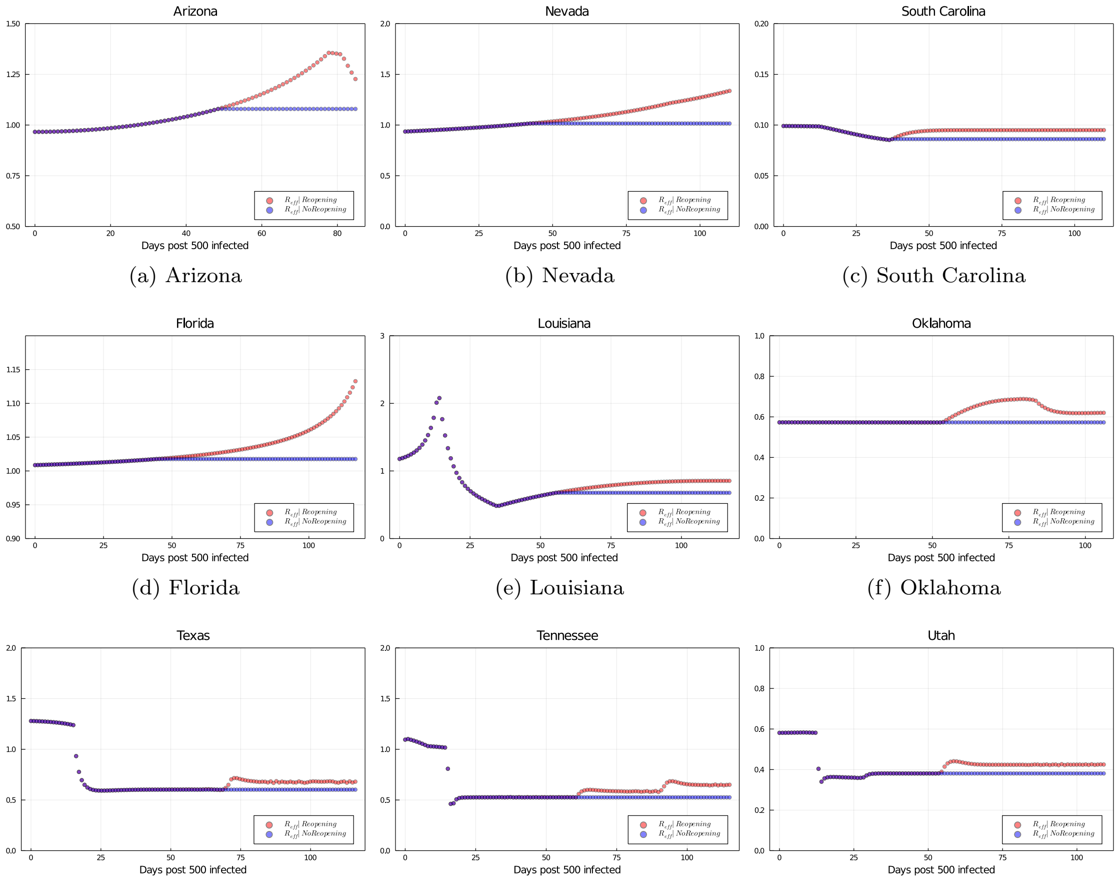

(a) Arizona (b) Nevada (c) South Carolina

(d) Florida (e) Louisiana (f) Oklahoma

(g) Texas (h) Tennessee (i) Utah

Figure S8: The comparison of the effective reproduction number, as defined in Equation 21 with and without reopening, shown for all US states considered in the present study

To further validate the Covid spread parameter variation and its relation to the effective reproduction number, we compare the variation in C_p to the R_{eff} obtained through a prominent Covid-19 forecasting model used by the CDC, USA.[7,8] For all of the 9 states which we considered, the time at which an upsurge is seen in C_p due to early reopening corresponds very well to the exact time at which an upsurge is seen in R_{eff}.[7,8] In addition, we show the comparison between C_p values estimated from our study and R_{eff} values obtained from[7,8] from reopening till one month post that; in table S2. For the states of Arizona, Nevada, Louisiana, Florida, Texas and Tennessee, these values lie close to each other. This further validates the results of our study and the quantitative metrics derived therein.

Region	C_p range (Our study)	R_{eff} range (Ref.[7])
Arizona	1.1 – 1.4	1.15 – 1.3
Florida	1 – 1.1	1.07 – 1.64
Nevada	1.05 – 1.5	1.19 – 1.5
Louisiana	0.7 – 1	0.88 – 1.62
Texas	0.6 – 0.7	1.08 – 1, 3
Tennessee	0.5 – 0.65	0.97 – 1.07

Table S2: C_p and R_{eff} value ranges from reopening till one month post that, for 6 states considered in our study; lie close to each other.

References

[1] Gillespie, D. T. (2000) The chemical langevin equation. The Journal of Chemical Physics 113, 297-306.

[2] McQuarrie, D. A. (1967) Stochastic approach to chemical kinetics. Cambridge University Press 4, 413-478.

[3] Gillespie, D. T. (1977) Exact stochastic simulation of coupled chemical reactions, The journal of physical chemistry 81, 2340-2361.

[4] Rasmussen, C. E. (2003) Gaussian processes in machine learning. Summer School on Machine Learning. 63-71.

[5] Williams, C. K & Rasmussen, C. E. (2006) Gaussian processes for machine learning. MIT Press.

[6] Dandekar, R, Rackauckas, C, & Barbastathis, G. (2020) A machine learning-aided global diagnostic and comparative tool to assess effect of quarantine control in covid-19 spread. Patterns 1, 100145.

[7] Gu, Y. (2021) COVID-19 Projections Using Machine Learning. (`https://covid19-projections.com/`).

[8] Roberts, S. (2021) Lessons from the pandemic's superstar data scientist, Youyang Gu. MIT Technology Review (`https://www.technologyreview.com/2021/04/27/1023657/lessons-from-the-pandemics-superstar-data-scientist-youyang-gu/`).
